# Analysis of Molecular Aspects of Periodontitis as a Risk Factor for Neurodegenerative Diseases: A Single-Center 10-Year Retrospective Cohort Study

**DOI:** 10.3390/ijms26062382

**Published:** 2025-03-07

**Authors:** Amr Sayed Ghanem, Marianna Móré, Attila Csaba Nagy

**Affiliations:** 1 Department of Health Informatics, Faculty of Health Sciences, University of Debrecen, 4032 Debrecen, Hungary; nagy.attila@etk.unideb.hu; 2 Institute of Social and Sociological Sciences, Faculty of Health Sciences, University of Debrecen, 4400 Nyíregyháza, Hungary; more.mariann@etk.unideb.hu

**Keywords:** periodontitis, neurodegenerative disease, cardiometabolic disease, cardiovascular disease, cerebrovascular disease, retrospective cohort, longitudinal, clinical, Weibull regression

## Abstract

Neurodegenerative diseases (NDDs) represent a considerable global health burden with no definitive treatments. Emerging evidence suggests that periodontitis may contribute to NDD through shared inflammatory, microbial, and genetic pathways. A retrospective cohort design was applied to analyze hospital records from 2012–2022 and to determine whether periodontitis independently increases NDD risk when accounting for major cardiovascular, cerebrovascular, metabolic, and inflammatory confounders. Likelihood ratio-based Cox regression tests and Weibull survival models were applied to assess the association between periodontitis and NDD risk. Model selection was guided by Akaike and Bayesian information criteria, while Harrell’s C-index and receiver operating characteristic curves evaluated predictive performance. Periodontitis demonstrated an independent association with neurodegenerative disease risk (HR 1.43, 95% CI 1.02–1.99). Cerebral infarction conferred the highest hazard (HR 4.81, 95% CI 2.90–7.96), while pneumonia (HR 1.96, 95% CI 1.05–3.64) and gastroesophageal reflux disease (HR 2.82, 95% CI 1.77–4.51) also showed significant increases in risk. Older individuals with periodontitis are at heightened risk of neurodegenerative disease, an effect further intensified by cerebrovascular, cardiometabolic, and gastroesophageal conditions. Pneumonia also emerged as an independent pathophysiological factor that may accelerate disease onset or progression. Attention to oral and systemic factors through coordinated clinical management may mitigate the onset and severity of neurodegeneration.

## 1. Introduction

Neurodegenerative diseases (NDDs) [1] constitute a critical global health concern, largely due to their progressive nature, substantial morbidity, and growing prevalence in aging populations [1]. Among these, Alzheimer’s disease (AD) and Parkinson’s disease (PD) are particularly prevalent, with AD affecting an estimated 6.2 million individuals aged 65 and older in the United States alone [2] and PD afflicting nearly one million Americans [3]. Worldwide, approximately 55 million people were living with dementia [4] in 2021, most commonly attributable to AD, a figure expected to rise to 139 million by 2050 [4]. Despite advances in supportive care, there remain no definitive cures or therapies that effectively halt the progression of these debilitating conditions.

Growing evidence from systematic reviews and observational studies suggests that periodontitis, defined as a chronic inflammatory disease of the oral cavity, may be linked to NDDs [5,6,7], particularly AD [8], through overlapping molecular pathways involving systemic inflammation, microbial invasion, and shared genetic susceptibilities. *Porphyromonas gingivalis*, a key pathogen implicated in periodontitis, has been detected in the brains of AD patients [8], where its virulence factors (gingipains) can breach the blood-brain barrier [9], provoke tau hyperphosphorylation, and foster amyloid-β aggregation [10]. Concurrently, elevated systemic levels of pro-inflammatory cytokines [6], such as Interleukin-1 beta (IL-1β), Interleukin-6 (IL-6), and Tumor Necrosis Factor-alpha (TNF-α), may amplify neuroinflammatory cascades and activate microglial cells, exacerbating neuronal injury. Genetic studies further indicate upregulated immune-related genes in both periodontitis and NDDs [6], hinting at convergent inflammatory pathways. Additionally, dysbiosis-induced disruptions in the oral–gut–brain axis can intensify systemic inflammation and contribute to neuropathology [6].

Despite considerable research linking periodontitis to NDDs, prior investigations have been constrained by limitations such as small cohorts, cross-sectional designs, and insufficient control for pivotal comorbid conditions. Additionally, many studies have focused exclusively on individual NDDs, such as Alzheimer’s or Parkinson’s disease, without analyzing them collectively. This narrow focus limits the understanding of potential shared mechanisms between periodontitis and various NDDs. Thus, a clear need remains for robust, long-term analyses to assess the independent contribution of periodontitis to NDD risk and elucidate potential underlying mechanisms. We hypothesized that periodontitis is independently associated with an increased risk of neurodegenerative diseases collectively, potentially mediated by inflammatory and microbial mechanisms. To address this, our single-center, 10-year retrospective cohort study employed multivariate Weibull models while accounting for major confounders such as cardiovascular, cerebrovascular, metabolic, and inflammatory conditions. Unlike previous research, this investigation integrated an extended follow-up period with rigorous analytical methods to isolate the specific contribution of periodontitis to NDD risk, thereby offering new insights into the molecular pathways underlying this association.

## 2. Results

### 2.1. Baseline Characteristics of the Study Participants

The study cohort, outlined in Table 1, included 4886 individuals with median age of 49 years (IQR: 38–60), with a range from 0 to 86 years. The cohort comprised 45.42% males (*n* = 2218) and 54.58% females (*n* = 2665). Periodontitis was present in 21.18% (*n* = 1035) of the cohort. Hypertension was reported in 12.53% (*n* = 612) of participants, while 6.88% (*n* = 336) had angina pectoris and 3.46% (*n* = 169) had chronic ischemic heart disease. Atrial fibrillation and heart failure were relatively uncommon, affecting 1.31% (*n* = 64) and 1.27% (*n* = 62) of participants, respectively. Cerebral infarction was observed in 0.53% (*n* = 26), and 1.11% (*n* = 54) had arterial occlusion or stenosis of the precerebral arteries. Other cerebrovascular diseases were present in 1.33% (*n* = 65), while atherosclerosis was diagnosed in 1.94% (*n* = 95). Non-toxic goiter was present in 2.33% (*n* = 114) of the participants. Diabetes and obesity affected 2.05% (*n* = 100) and 2.42% (*n* = 118) of the cohort, respectively. Disorders of lipoprotein metabolism were observed in 6.06% (*n* = 296). Pneumonia was recorded in 1.39% (*n* = 68), and 1.53% (*n* = 75) had gastroesophageal reflux disease.

### 2.2. Cumulative Hazard Analysis Using Nelson–Aalen Estimates

**Figure 1:**
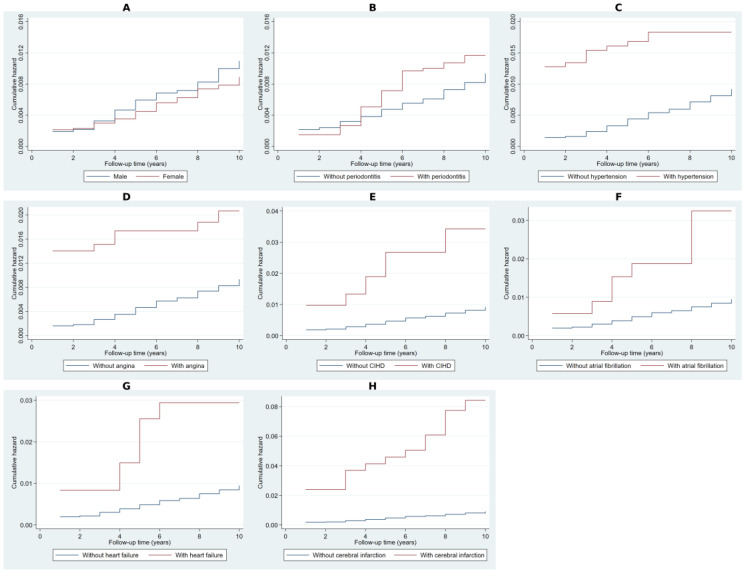
Cumulative hazard estimates for risk factors using Nelson–Aalen analysis. Note: Panel (**A**) presents the cumulative hazard estimates comparing males and females. Panel (**B**) illustrates differences between individuals with and without periodontitis. Panel (**C**) shows the cumulative hazard for hypertension, while Panel (**D**) depicts angina pectoris. Panel (**E**) represents chronic ischemic heart disease. Panels (**F**,**G**) display cumulative hazard estimates for atrial fibrillation and heart failure, respectively. Panel (**H**) presents the cumulative hazard for cerebral infarction.

The Nelson–Aalen cumulative hazard estimates in Figure 1 demonstrated a higher cumulative hazard for NDD over time among individuals with key risk factors compared to those without. Periodontitis was associated with an increased cumulative hazard, though the divergence remained modest. More pronounced risk differentials were observed for hypertension, angina pectoris, and chronic ischemic heart disease, where affected individuals exhibited consistently higher cumulative hazard trajectories throughout the follow-up period. The greatest separation in hazard accumulation was observed in individuals with atrial fibrillation, heart failure, and cerebral infarction, indicating a significantly elevated risk of NDD over time.

**Figure 2:**
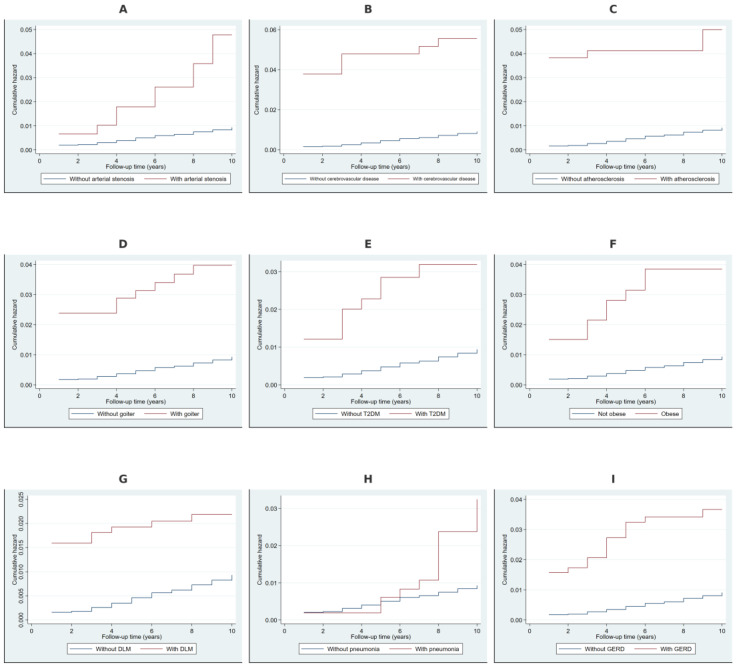
Nelson–Aalen cumulative hazard estimates for additional risk factors. Note: Panel (**A**) presents the cumulative hazard estimates for individuals with and without arterial stenosis. Panel (**B**) compares individuals with and without cerebrovascular disease, while Panel (**C**) illustrates the cumulative hazard associated with atherosclerosis. Panel (**D**) shows hazard estimates for non-toxic goiter, and Panel (**E**) for type 2 diabetes mellitus (T2DM). Panel (**F**) depicts the cumulative hazard for obesity. Panel (**G**) examines disorders of lipoprotein metabolism (DLM), while Panels (**H**,**I**) compare cumulative hazard estimates for pneumonia and gastroesophageal reflux disease (GERD), respectively.

The Nelson–Aalen cumulative hazard estimates in Figure 2 demonstrated a significantly higher cumulative hazard for NDD over time among individuals with arterial stenosis, cerebrovascular disease, and atherosclerosis, indicating an elevated long-term risk. Individuals with non-toxic goiter and type 2 diabetes mellitus exhibited a moderately increased cumulative hazard, with a more gradual divergence from those without these conditions. Obesity, disorders of lipoprotein metabolism, pneumonia, and gastroesophageal reflux disease were also associated with a steeper increase in cumulative hazard over time, suggesting their contribution to an increased risk of developing NDD.

### 2.3. Cox Regression-Based Likelihood Ratio Tests for Covariates

In univariate Cox regression analysis (Table 2), periodontitis was associated with an increased relative hazard of 1.31 (*p* = 0.064) for NDD risk, though it did not reach statistical significance. Hypertension showed a significantly higher risk, with a relative hazard of 2.47 (*p* < 0.001). Angina pectoris and chronic ischemic heart disease were also significantly associated with increased NDD risk, with relative hazards of 2.6 (*p* < 0.001) and 4.16 (*p* < 0.001), respectively. Atrial fibrillation (3.62, *p* = 0.002) and heart failure (3.73, *p* = 0.001) were both strong predictors. Cerebral infarction exhibited the highest relative hazard at 9.84 (*p* < 0.001), followed by arterial occlusion or stenosis of the precerebral arteries (5.01, *p* < 0.001), other cerebrovascular diseases (6.91, *p* < 0.001), and atherosclerosis (6.22, *p* < 0.001). Non-toxic goiter (4.82, *p* < 0.001), diabetes (4.00, *p* < 0.001), and obesity (4.79, *p* < 0.001) were also significant predictors of NDD risk. Disorders of lipoprotein metabolism (2.76, *p* < 0.001), pneumonia (2.73, *p* = 0.004), and gastroesophageal reflux disease (4.53, *p* < 0.001) were significantly associated with an increased hazard of developing NDD. Gender was not significantly associated with NDD risk (*p* = 0.132).

### 2.4. Kaplan–Meier Survival Estimates Stratified by Covariates

**Figure 3:**
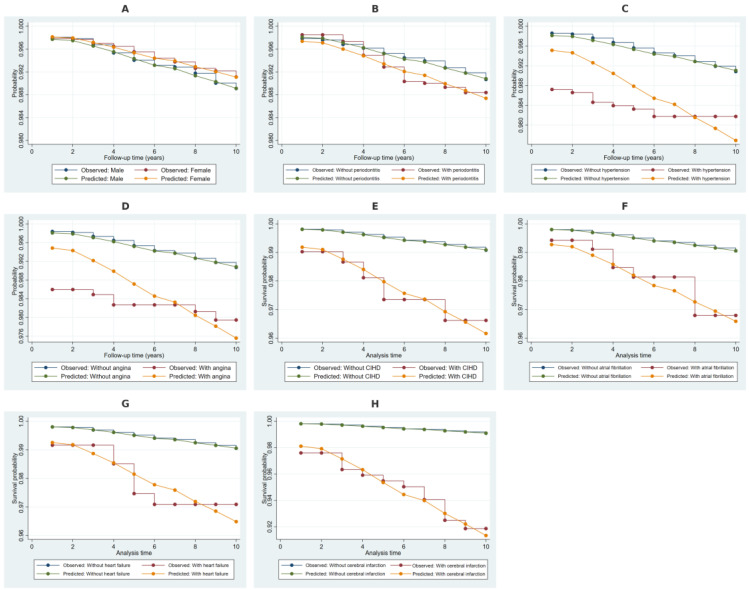
Kaplan–Meier survival estimates with Cox model predictions for NDD. Note: Panel (**A**) presents the Kaplan–Meier observed survival estimates and Cox model-predicted survival probabilities for males and females. Panel (**B**) illustrates survival estimates for individuals with and without periodontitis. Panels (**C**–**E**) show survival estimates for hypertension, angina pectoris, and chronic ischemic heart disease (CIHD), respectively. Panels (**F**–**H**) display survival trajectories for individuals with and without atrial fibrillation, heart failure, and cerebral infarction, respectively. Both observed and predicted survival probabilities are included to assess the model’s fit in stratifying NDD risk across these covariates.

The Kaplan–Meier survival estimates, along with Cox model-predicted survival probabilities in Figure 3, demonstrate differential survival trajectories for NDD based on covariate stratification. The observed and predicted survival probabilities for males and females showed minimal divergence over time. Individuals with periodontitis exhibited slightly lower survival probabilities compared to those without, though the difference remained small. More pronounced survival differences were observed in individuals with hypertension, angina pectoris, and chronic ischemic heart disease, where affected individuals consistently showed lower survival probabilities. The greatest separation in both observed and predicted survival was evident among individuals with atrial fibrillation, heart failure, and cerebral infarction, indicating a significantly elevated NDD risk in these groups.

**Figure 4:**
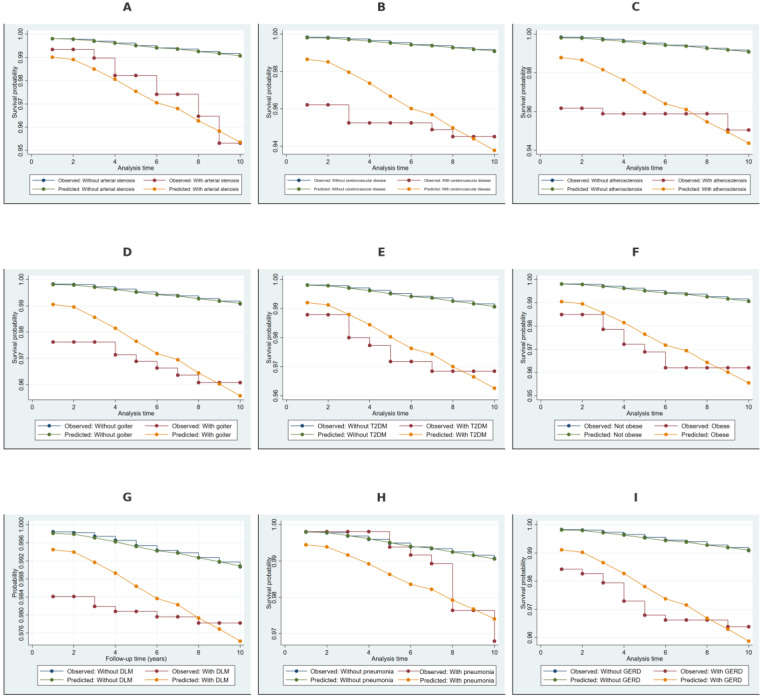
Kaplan–Meier survival estimates with Cox model predictions for NDD (additional covariates). Note: Panel (**A**) presents Kaplan–Meier observed survival estimates and Cox model-predicted survival probabilities for individuals with and without arterial stenosis. Panel (**B**) illustrates survival estimates for cerebrovascular disease, while Panel (**C**) shows survival stratification for atherosclerosis. Panels (**D**,**E**) display survival estimates for non-toxic goiter and type 2 diabetes mellitus (T2DM), respectively. Panel (**F**) presents survival estimates for obesity. Panel (**G**) examines survival stratification by disorders of lipoprotein metabolism (DLM), while Panels (**H**,**I**) compare survival probabilities for pneumonia and gastroesophageal reflux disease (GERD), respectively. Observed and predicted survival estimates are included to assess the model’s performance in predicting NDD risk across these covariates.

The Kaplan–Meier survival estimates with Cox-based model predictions in Figure 4 showed a significantly lower survival probability for NDD among individuals with arterial stenosis, cerebrovascular disease, and atherosclerosis, with a clear separation from those without these conditions. Non-toxic goiter and type 2 diabetes mellitus were associated with moderately lower survival probabilities, with a more gradual divergence over time. Individuals with obesity, disorders of lipoprotein metabolism, pneumonia, and gastroesophageal reflux disease exhibited a steeper decline in survival probabilities, indicating a higher cumulative risk of NDD.

### 2.5. Weibull Regression Analysis of Risk Factors for NDD

**Figure 5:**
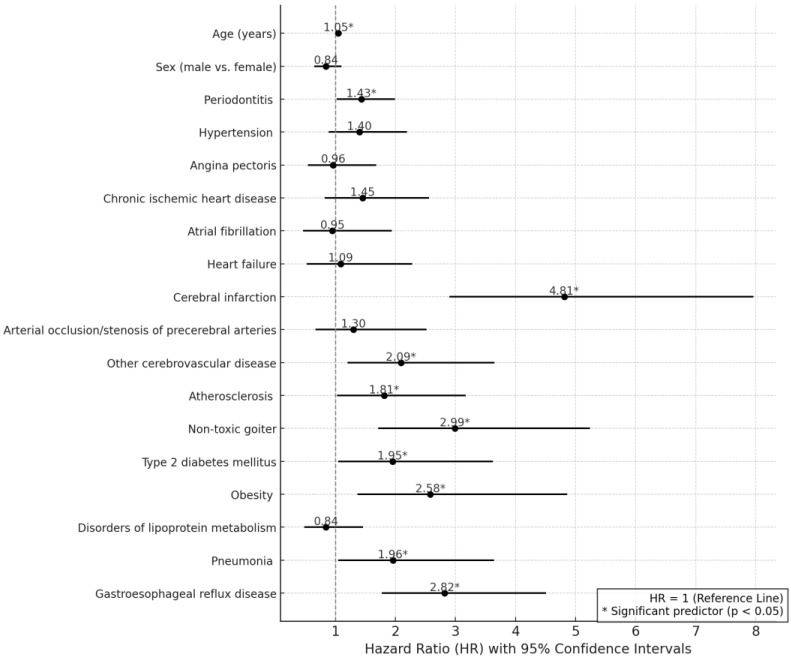
Forest plot of hazard ratios for neurodegenerative disease risk based on results of multivariable Weibull regression model. Note: Significant predictors (*p* < 0.05) are marked with an asterisk (*). The vertical dashed line represents the hazard ratio (HR) reference value of 1, indicating no effect. Horizontal lines extending from each point estimate denote the 95% confidence intervals, reflecting the range within which the true HR is expected to lie with 95% certainty.

In the Weibull regression analysis, results in Table 3 and Figure 5 indicated that increasing age was significantly associated with a higher hazard of NDD (HR = 1.05, 95% CI: 1.04–1.07, *p* < 0.001). Periodontitis was a significant predictor, increasing the hazard of NDD by 43% (HR = 1.43, 95% CI: 1.02–1.99, *p* = 0.037). Cerebral infarction exhibited the highest hazard, more than quadrupling the risk of NDD (HR = 4.81, 95% CI: 2.90–7.96, *p* < 0.001). Other cerebrovascular diseases (HR = 2.09, 95% CI: 1.20–3.65, *p* = 0.01), atherosclerosis (HR = 1.81, 95% CI: 1.03–3.17, *p* = 0.038), non-toxic goiter (HR = 2.99, 95% CI: 1.71–5.24, *p* < 0.001), diabetes (HR = 1.95, 95% CI: 1.05–3.62, *p* = 0.034), obesity (HR = 2.58, 95% CI: 1.37–4.86, *p* = 0.003), pneumonia (HR = 1.96, 95% CI: 1.05–3.64, *p* = 0.035), and gastroesophageal reflux disease (HR = 2.82, 95% CI: 1.77–4.51, *p* < 0.001) were also significant predictors of NDD. Hypertension, angina pectoris, chronic ischemic heart disease, atrial fibrillation, heart failure, and arterial stenosis did not show significant associations. Gender was not significantly associated with NDD risk (HR = 0.84, 95% CI: 0.65–1.10, *p* = 0.204).

### 2.6. Discriminative Performance of the Weibull Model

**Figure 6:**
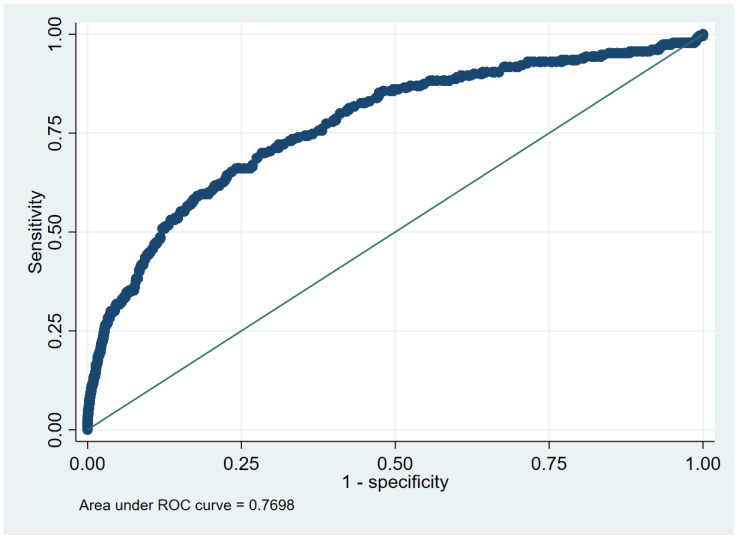
Area under the receiver operating characteristic curve for the Weibull model.

The receiver operating characteristic (ROC) curve analysis, presented in Figure 6, evaluated the discriminatory ability of the Weibull model in predicting NDD risk. The area under the curve (AUC) was 0.77 (95% CI: 0.74–0.80), indicating good to excellent predictive performance. The standard error of the AUC was 0.02, reflecting a relatively precise estimate. The curve deviates substantially from the diagonal reference line, suggesting that the model provides meaningful differentiation between individuals with and without NDD. These findings support the model’s robustness in capturing risk patterns associated with NDD development.

## 3. Discussion

The current study is the first to evaluate periodontitis as a risk factor for multiple neurodegenerative diseases concurrently using Weibull survival analysis, while also incorporating pneumonia as a novel factor in the risk assessment, alongside cardiovascular, metabolic, and inflammatory comorbidities. The Weibull regression analysis demonstrated that periodontitis was a significant independent predictor of neurodegenerative disease risk. Among cerebrovascular conditions, cerebral infarction exhibited the strongest association, while atherosclerosis, type 2 diabetes mellitus (T2DM), obesity, pneumonia, and gastroesophageal reflux disease (GERD) also contributed significantly.

Aging emerged as a significant risk factor for NDDs in this study, consistent with prior findings [11,12]. The heightened susceptibility of postmitotic cells, such as neurons and oligodendrocytes, to age-related molecular disruptions contributes to this increased risk [13,14]. One major contributor is epigenetic dysregulation [15], particularly aberrant DNA methylation mediated by DNA methyltransferases, which can disrupt neuronal gene expression and compromise genomic stability. Accumulation of DNA damage [16] further exacerbates this instability, promoting neurodegeneration in conditions such as Alzheimer’s and Parkinson’s disease. Additionally, mitochondrial dysfunction, including declining nicotinamide adenine dinucleotide (NAD+) levels, impairs cellular metabolism and proteostasis, leading to increased oxidative stress and energy deficits [17]. Cellular senescence, telomere attrition, and chronic inflammatory signaling further contribute to neuronal decline, creating a cascade of interconnected processes that accelerate neurodegeneration.

Beyond intrinsic aging-related mechanisms, external inflammatory stimuli may further accelerate neurodegenerative processes. Periodontitis, a chronic inflammatory condition of the oral cavity, represents one such factor, and its significant association with NDD risk in this study further strengthens its potential role in disease progression. Periodontitis, characterized by gram-negative bacterial infection [18] (e.g., *Porphyromonas gingivalis*) and persistent gingival inflammation, may initiate or exacerbate neurodegenerative processes by fueling low-grade systemic inflammation and promoting the translocation of oral pathogens and their virulence factors into systemic circulation [7]. *P. gingivalis* releases gingipains, proteolytic enzymes capable of degrading immune receptors such as CD14, which may dampen host defenses against lipopolysaccharides (LPS) and potentiate inflammatory signaling cascades [5]. The chronic rupture of periodontal pockets creates an accessible route for pathogenic bacteria and pro-inflammatory cytokines—including IL-1β, TNF-α, and IL-6—to enter the bloodstream [19], contributing to elevated inflammatory mediators that can cross the blood–brain barrier and induce microglial activation [5]. Microglia, upon sensing these systemic stimuli, shift toward a pro-inflammatory phenotype that produces neurotoxic levels of reactive oxygen species and cytokines, potentially accelerating neuronal damage. IL-8 dysregulation also appears critical, since *P. gingivalis* gingipains may degrade IL-8, altering the delicate balance of immune responses in both peripheral and central compartments [6]. Risk stratification studies suggest a bidirectional interplay in which individuals with neurodegenerative diseases frequently exhibit reduced oral hygiene capacity, exacerbating periodontal pathology [6,20].

The current findings expanded on existing literature by analyzing periodontitis in relation to multiple neurodegenerative outcomes within a 10-year survival framework, integrating rigorous survival modeling and comprehensive adjustment for comorbidities. Unlike previous studies that primarily examined individual NDDs, this study offers a broader epidemiological perspective on how chronic periodontal inflammation may influence diverse neurodegenerative pathways.

In addition to periodontal inflammation, cerebrovascular pathology emerged as a significant risk factor for neurodegenerative diseases in this study. Cerebral infarction exhibited the strongest association, while other cerebrovascular conditions also contributed significantly. Emerging research [21,22,23] highlights cerebrovascular abnormalities as pivotal contributors to neurodegenerative disease pathology. Neuronal loss remains a defining feature of conditions such as Alzheimer’s disease (AD), amyotrophic lateral sclerosis (ALS), and Huntington’s disease (HD), yet vascular disruptions often precede or exacerbate this neuronal decay [24,25]. Alterations in the blood–brain barrier (BBB), including reduced pericyte number and function [26], can impair clearance of toxic proteins like amyloid-β and enable infiltration of immune cells that intensify inflammatory cascades. Chronic reductions in cerebral blood flow [27] and morphological changes in capillaries [28] may further compromise neuronal viability, while genotypic variations—such as the APOE4 allele—amplify vascular fragility and pericyte vulnerability [29]. These interconnected deficits can lead to microhemorrhages, capillary constriction, or white matter lesions, ultimately aggravating cognitive decline and accelerating disease progression [30]. A recurring theme in multiple neurodegenerative disorders is the loss of vascular integrity, which fosters a pro-inflammatory environment that has profound implications for both the onset and evolution of pathology in the central nervous system.

Given the critical role of vascular integrity in neurodegeneration, this study further identified atherosclerosis, type 2 diabetes mellitus, and obesity as significant risk factors. These conditions, closely linked to metabolic and vascular dysfunction, may play an important role in exacerbating neurodegenerative processes. All three aforementioned conditions converge on pathological pathways that disrupt cerebrovascular integrity, heighten systemic and neural inflammation, and compromise metabolic homeostasis, ultimately fostering neurodegenerative processes. Atherosclerotic lesions initiate with endothelial dysfunction that weakens the blood–brain barrier [31], permitting leukocyte infiltration and escalating oxidative injury. This dysfunction compromises the neurovascular unit, impairing clearance of neurotoxic proteins such as amyloid-β and tau [31], thereby predisposing the brain to toxic aggregates. T2DM augments the effects of vascular damage through hyperglycemia, hyperinsulinemia, and insulin resistance [32], which together accelerate brain atrophy and intensify risk for dementia. Dysregulated insulin signaling also impairs amyloid-β degradation via insulin-degrading enzyme competition [33], fueling cerebral accumulation of amyloid-β. Obesity further amplifies these threats by perpetuating low-grade inflammation, altering adipokine secretion, and diminishing mitochondrial efficiency [34]. These metabolic imbalances exacerbate insulin resistance and worsen endothelial cell activation, reinforcing the cycle of neuroinflammation and neuronal dysfunction [34]. Taken together, these factors generate a milieu that erodes both vascular and neuronal resilience, thereby linking atherosclerosis, T2DM, and obesity to neurodegenerative disease pathology.

Despite its classification as a benign condition without overt thyroid dysfunction, non-toxic goiter has not previously been explored as a potential contributor to neurodegenerative risk. The observed association in our study may be mediated through subtle thyroid inflammation, iodine dysregulation, or vascular alterations that heighten oxidative stress and endothelial dysfunction—core processes implicated in neurodegeneration [17,35]. Non-toxic goiter shares key risk factors with neurodegenerative diseases, such as advanced age [36], metabolic syndrome [37], and cardiovascular disease [38], suggesting a more systemic pathophysiological interplay rather than a direct causal mechanism. Given the limited research on this association, further studies are needed to determine whether non-toxic goiter contributes directly to neurodegeneration or reflects underlying systemic processes.

While vascular and metabolic disturbances are increasingly recognized as contributors to neurodegeneration, the potential role of gastrointestinal pathology remains less explored. In this study, GERD emerged as a significant predictor of neurodegenerative disease risk, raising important questions about the neurological consequences of chronic esophageal inflammation and recurrent microaspiration. GERD has been implicated in a heightened risk of neurodegenerative disorders through mechanisms involving sustained inflammation and altered gut–brain axis interactions. Esophageal epithelial damage from repeated exposure to gastric acid can elicit chemokine production and immune cell recruitment [39], raising local and systemic levels of proinflammatory mediators shown to correlate with accelerated cognitive decline. Perturbations in normal vagal and spinal innervation may further contribute to neuronal dysfunction [40], while discrepancies in proton-pump inhibitor (PPI) research emphasize the complexity of pharmacological influences on neurodegeneration. Some evidence suggests that PPIs can modulate amyloid protein processing [41], although clinical data remain inconclusive regarding their direct impact on dementia risk.

Neuroinflammatory pathways linked to gastrointestinal dysfunction may not be confined to the gut alone but could extend to the respiratory system, where chronic and recurrent infections further exacerbate neurodegenerative processes. In this study, pneumonia was identified as a significant predictor of neurodegenerative disease risk, marking a lesser-explored yet biologically plausible contributor to neurodegeneration. While prior research has largely focused on systemic inflammation and vascular dysfunction, the potential role of respiratory infections in shaping neurodegenerative trajectories remains underexamined, warranting further investigation into their pathophysiological mechanisms. Pneumonia could be a potential driver of neurodegenerative pathogenesis by initiating systemic inflammatory responses that can breach the BBB and trigger pathological processes within the central nervous system [42]. Acute pulmonary infections often result in elevated circulating levels of IL-6, IL-8, and TNF-α, which may enter the central circulation or activate afferent neural pathways [42], leading to microglial and astrocytic priming. Experimental data indicate that severe pneumonia can disrupt the lung-blood barrier and BBB simultaneously, facilitating bacterial translocation into the brain and further heightening neuronal injury [43]. Epidemiological investigations lend additional support, showing that patients hospitalized for pneumonia are at increased risk of subsequent cognitive decline or dementia across various age groups [43]. Although individuals with established neurodegenerative disorders frequently display risk factors for pneumonia [44], such as diminished airway protection and reduced mobility, our findings underscore the possibility that pneumonia itself may serve as an independent pathophysiological insult, capable of accelerating or even initiating neurodegenerative processes through systemic inflammation and direct microbial impacts on the brain. While pneumonia demonstrated a significant association with neurodegenerative disease risk, its impact size in the survival model was more modest compared to cerebrovascular pathology and metabolic disorders, suggesting its role as an exacerbating factor rather than a primary driver.

### Strengths and Limitations

This investigation draws on real-world clinical data spanning a 10-year follow-up period, which enables a comprehensive view of how periodontitis may evolve alongside neurodegenerative outcomes under usual clinical conditions. Diagnoses based on ICD-10 codes assure clinical relevance, and the large cohort size provides robust statistical power. Employing a longitudinal, time-to-event analytical framework (including Weibull survival models) allows for the adjustment of a broad spectrum of comorbidities and potential confounders, thereby affording a more nuanced understanding of the temporal and causal associations between periodontitis, systemic comorbidities and neurodegenerative diseases. Limitations stem from the study using data from a single center; thus, the external validity of the findings may be constrained. Reliance on ICD-10 codes does not capture the full clinical severity or staging of either periodontitis or neurodegenerative conditions, potentially obscuring more granular associations. Missing sociodemographic variables precludes exploration of their contributory roles, while the absence of smoking status presents a confounding factor that cannot be fully controlled. The retrospective study design carries inherent biases in data recording and availability, thereby limiting the analysis of these parameters. Moreover, as an observational study, there remains a possibility that unmeasured factors could influence the observed relationships, and causality cannot be definitively established. Another inherent limitation of registry-based data is that there is a small likelihood of cases diagnosed outside the hospital system.

## 4. Materials and Methods

### 4.1. Database Cleaning and Data Processing

This hospital-based retrospective cohort study [45] was conducted as a secondary analysis of data from the University of Debrecen Hospital, covering the period from 2007 to 2022. The initial dataset comprised 37,164 recorded clinical encounters; however, data prior to 2012 were excluded due to a high proportion of missing values and incomplete documentation of periodontitis, ensuring consistency in disease ascertainment and minimizing information bias. After data refinement, the final dataset included 4886 unique patients, contributing a total of 29,918 recorded encounters over the follow-up period. The presence or absence of NDD and other comorbid conditions was determined based on International Classification of Diseases, 10th Revision (ICD-10) codes [46], systematically recorded by hospital-affiliated physicians across relevant medical departments, while dental-related ICD-10 codes were specifically diagnosed and documented by dental practitioners within the hospital’s dental units to ensure diagnostic accuracy. Follow-up time was defined from each patient’s earliest recorded hospital encounter until NDD diagnosis, while individuals who did not develop NDD were censored at their last recorded hospital visit or at the study endpoint in 2022, assuming non-informative censoring. This approach ensured a robust time-to-event analysis framework, allowing for accurate estimation of disease occurrence and progression.

### 4.2. Inclusion and Exclusion Criteria

To ensure a disease-free cohort at baseline, individuals with a documented diagnosis of any neurodegenerative disease were excluded during or prior to the first year of follow-up, preventing the inclusion of prevalent cases and mitigating survival bias. Participants were included if they had at least two recorded hospital encounters during the study period and a minimum follow-up duration of one year, defined as the time between the first recorded visit and either NDD diagnosis or censoring. This threshold ensured that all included individuals had adequate longitudinal observation for meaningful survival analysis.

Exclusion criteria encompassed individuals with incomplete or missing diagnostic records for periodontitis, as accurate exposure classification was essential for robust risk estimation. The exclusion of individuals with missing periodontitis data, while necessary for methodological rigor, introduces a potential selection bias if missingness was not random. Therefore, baseline characteristics of excluded and retained participants were compared to assess potential systematic differences. Individuals with only a single recorded hospital visit and no subsequent encounters were also excluded, as they lacked sufficient follow-up time to contribute valid person-time in the survival analysis framework, and their inclusion could introduce immortal time bias.

Participants who did not develop NDD by the end of the study period were censored at their last recorded hospital visit or at the study endpoint in 2022, whichever occurred first. Individuals lost to follow-up before the study endpoint were retained in the analysis and censored at their last recorded visit, assuming non-informative censoring, meaning that loss to follow-up was considered independent of the likelihood of developing NDD. Given that hospital-based administrative data have high sensitivity for capturing NDD diagnoses due to the chronic and progressive nature of these conditions, the likelihood of undiagnosed cases outside the hospital system is expected to be low [47,48], though it remains a potential limitation. This approach ensured that all included individuals were initially free of NDD, had sufficient longitudinal follow-up, and possessed complete exposure and outcome data, adhering to the methodological aspects required for retrospective cohort analyses.

### 4.3. Variable Definitions and Covariate Selection

NDDs were defined based on the presence of specific ICD-10 codes, as recorded in the hospital’s electronic health system. The following diagnostic codes were used to classify NDD cases: G11 (Hereditary ataxias), G12 (Spinal muscular atrophy and related syndromes), G20 (Parkinson’s disease), G21–G22 (Parkinsonian syndromes), G30 (Alzheimer’s disease), G31 (Other degenerative diseases of the nervous system), F01–F03 (Vascular and unspecified dementias), and G93 (Other disorders of the brain).

Huntington’s disease (G10) and other degenerative diseases of the basal ganglia (G23) were not included in the analysis, as there were no recorded cases within the dataset. Their absence did not impact the model’s performance or validity, as no observations were lost due to missing categories.

Periodontitis was identified using ICD-10 codes K05, K05.2, K05.3, K05.4, K05.5, and K05.6, which encompass various clinical manifestations of periodontal disease, including aggressive periodontitis, chronic periodontitis, and other specified periodontal conditions.

The selection of covariates was based on established epidemiological and mechanistic associations with both periodontitis and NDDs, ensuring appropriate confounder control. Age [14] and sex [49] were included as fundamental demographic confounders, given their strong influence on NDD incidence and periodontitis prevalence [50]. Hypertension, ischemic heart disease [51], cerebral infarction, other cerebrovascular diseases [21], and atherosclerosis [31] were incorporated as major vascular risk factors that contribute to neurodegeneration through mechanisms such as chronic hypoperfusion, endothelial dysfunction, and increased amyloid deposition [35]. Type 2 diabetes [52] and obesity [34] were included due to their role in systemic inflammation and metabolic dysregulation, both of which accelerate neurodegeneration and exacerbate periodontitis [53]. Hyperlipidemia was included as a potential confounder, as dyslipidemia independently contributes to both atherosclerosis-associated neurodegeneration [54] and inflammatory responses that exacerbate periodontitis [55], meeting the criteria for confounding. Pneumonia and GERD were included as confounders due to their independent associations with both periodontitis and NDD. Pneumonia contributes to systemic inflammation and frailty [42,56], while GERD reflects chronic inflammatory processes that increase susceptibility to both conditions [57,58]. Importantly, neither condition lies within the causal pathway linking periodontitis to neurodegeneration, ensuring proper adjustment. Despite comprehensive confounder selection, residual confounding due to unmeasured variables cannot be fully excluded, though the inclusion of major cardiometabolic, inflammatory, and vascular risk factors mitigates this concern.

### 4.4. Statistical Methods

Baseline characteristics were derived from each participant’s first recorded hospital encounter within the study period. The distribution of continuous variables was assessed for normality using the Shapiro–Wilk test [59]. Normally distributed variables were summarized using means and standard deviations (SD), while non-normally distributed variables were reported as medians with interquartile ranges (IQR). Categorical variables were described as frequencies and proportions.

#### 4.4.1. Cumulative Hazard Estimation

Cumulative hazard estimates were derived using the Nelson–Aalen estimator [60], a non-parametric approach that provides a stepwise estimate of the cumulative hazard function over time. This method is particularly suited for right-censored survival data, as it accounts for varying follow-up durations and does not require assumptions regarding the underlying hazard distribution. The Nelson–Aalen estimator was computed for each stratified covariate to assess differential hazard accumulation across exposure groups.

To facilitate interpretability, cumulative hazard functions were visualized graphically, allowing for a comparative assessment of hazard trajectories among individuals with and without periodontitis and other comorbid conditions. This approach aids in identifying potential deviations in hazard accumulation patterns, which may reflect underlying differences in disease progression dynamics. The rationale for employing the Nelson–Aalen estimator in the present analysis lies in its robustness to censoring and its ability to provide an unbiased estimate of cumulative hazard, offering a preliminary understanding of the time-dependent risk associated with NDD development before proceeding to multivariable survival modeling. This estimator is defined as follows:

(1) $$\hat{H}\left(t\right)=\underset{t_{i}\leq t}{\sum }\frac{d_{i}}{n_{i}}$$

where $\hat{H}\left(t\right)$ is the cumulative hazard at time t, $d_{i}$ represents the number of events (failures) at time $t_{i}$ and $n_{i}$ denotes the number of individuals at risk immediately before $t_{i}$.

Nelson–Aalen cumulative hazard estimates were included to quantify absolute risk accumulation over time, providing clinically relevant information on disease progression. Unlike Kaplan–Meier survival probabilities, cumulative hazard estimates illustrate the intensity of risk over follow-up, aiding in risk stratification and improving the interpretation of long-term disease burden.

This approach was also useful for identifying deviations in hazard trajectories across exposure groups, which informed our assessment of proportionality assumptions. Additionally, the observed hazard patterns supported the transition from Cox regression to the Weibull survival model by highlighting the non-constant hazard function, further justifying the choice of a parametric approach for improved model fit and predictive accuracy.

#### 4.4.2. Likelihood Ratio Test for Equality of Survival Distributions

The univariate, Cox regression-based likelihood ratio (LR) test for equality of survival functions was employed to assess whether the survival distributions differed significantly across categorical covariate strata [61]. This test evaluates the null hypothesis that the survival functions are identical between comparison groups by quantifying the improvement in model fit when the covariate of interest is included. The LR test statistic follows a chi-square distribution and is derived by comparing the log-likelihoods of nested models, specifically a restricted model excluding the covariate and a full model incorporating it.

The rationale for its application in the present analysis stems from its superiority over the log-rank test when event times are tied, as it is less sensitive to violations of the proportional hazards assumption and provides a likelihood-based assessment of survival differences. By leveraging this approach, we ensured a statistically rigorous comparison of survival distributions across exposure categories, identifying variables that warranted further investigation in multivariable survival models.

Calculation of the likelihood ratio in the context of proportional hazard regression is based on the comparison of the log-likelihoods of two nested models:

- Restricted Model ($L_{0}$): A model without the covariate of interest.
- Full Model ($L_{1}$): A model including the covariate of interest.

The test statistic is calculated as follows:

(2) $$LR=-2(logL_{0}-logL_{1})$$

where $L_{0}$ is the likelihood of the restricted model (null hypothesis: no effect of the covariate) and $L_{1}$, which is the likelihood of the full model (alternative hypothesis: the covariate influences survival).

The resulting LR statistic follows an asymptotic chi-square ($X^{2}$) distribution with degrees of freedom equal to the difference in the number of parameters between the two models. The *p*-value is obtained by comparing the LR statistic to the chi-square distribution, determining whether the inclusion of the covariate significantly improves model fit.

#### 4.4.3. Kaplan–Meier Estimates

To assess the concordance between observed survival probabilities and those predicted by the Cox proportional hazards model, Kaplan–Meier survival estimates [62] were plotted alongside model-derived predicted survival curves. This approach facilitates a visual assessment of model fit by comparing non-parametric empirical survival probabilities with semi-parametric Cox regression estimates across different covariate strata.

The Kaplan–Meier estimator is a non-parametric function that estimates survival probability at time t as follows:

(3) $$\hat{s}\left(t\right)=\underset{t_{i}\leq t}{\prod }\left(1-\frac{d_{i}}{n_{i}}\right)$$

where:

- $\hat{s}\left(t\right)$ is the estimated survival probability at time t,
- $d_{i}$ is the number of events (e.g., diagnosis of periodontitis) at time $t_{i}$,
- $n_{i}$ is the number of participants at risk just before time $t_{i}$.

In contrast, Cox model-based predicted survival is obtained by incorporating estimated hazard ratios into the baseline survival function:

(4) $$\hat{S}\left(t|X\right)=S_{0}(t{)}^{exp(\beta X)}$$

where:

- $S_{0}(t)$ is the baseline survival function,
- β is the vector of estimated regression coefficients,
- X is the covariate matrix.

#### 4.4.4. Multivariable Parametric Survival Modeling Using the Weibull Regression Model

While the Cox proportional hazards model provides a robust semi-parametric approach for survival analysis without assuming an explicit baseline hazard function, its reliance on the proportional hazards (PH) assumption [63] necessitates rigorous validation. Initial model diagnostics were performed using Schoenfeld residuals, which revealed a violation of the PH assumption for the age variable, indicating non-proportionality over time. Given that age is a fundamental predictor of NDD onset, this violation raised concerns regarding the suitability of the Cox model for accurately capturing time-dependent hazard dynamics in the multivariate model.

To further assess model performance, we compared the Akaike Information Criterion (AIC) and Bayesian Information Criterion (BIC) between the Cox and parametric alternatives [64]. The Weibull regression model demonstrated a substantially lower AIC and BIC, indicating superior model fit. Additionally, the area under the receiver operating characteristic curve (AUC) [65] and Harrell’s concordance index (C-index) [66] were computed for both models, with the Weibull model yielding significantly higher discrimination ability, further supporting its selection.

The Weibull regression model assumes a parametric hazard function in the following form:

(5) $$h\left(t|X\right)=\lambda pt^{p-1}e^{\beta X}$$

where:

- $h\left(t|X\right)$ is the hazard function at time t given covariates X;
- λ is the scale parameter;
- p is the shape parameter, which determines whether the hazard is increasing (p>1), constant (p=1), or decreasing (p<1) over time;
- βX represents the linear predictor derived from the covariate effects.

Unlike the Cox model, which assumes an unspecified baseline hazard, the Weibull model [67] provides a fully specified hazard function, allowing for explicit modelling of time-dependent risk dynamics. This feature is particularly advantageous given the age-related acceleration of NDD risk, which is better captured by the Weibull’s flexible hazard structure. The significantly improved model fit and predictive accuracy, alongside violations of the proportional hazard assumption in Cox regression, justified the use of Weibull regression as the preferred survival model in this analysis. All model estimates were expressed as adjusted hazard ratios (HR) with corresponding 95% confidence intervals (CI), providing a quantifiable measure of relative risk while accounting for covariate effects. The level of statistical significance was set at *p* < 0.05. All analyses, including model estimation, diagnostic assessments, and graphical visualizations, were conducted using Stata version 18 [68]. The forest plot was generated in Python version 3.13.1 using the Matplotlib package version 3.9.0 [69].

## 5. Conclusions

Older individuals presenting with periodontitis appear to face an elevated risk of developing neurodegenerative diseases, highlighting the potential impact of oral inflammation on brain health. The presence of cerebrovascular and cardiometabolic comorbidities, as well as gastroesophageal reflux disease, may further compound neurodegenerative risk, prompting vigilant screening for early neurological symptoms. Pneumonia, in turn, emerged as a plausible independent pathophysiological factor that could exacerbate or accelerate neurodegenerative trajectories. Coordinated efforts at primary, secondary, and tertiary prevention may help reduce the cumulative risk burden and slow disease progression, allowing clinicians greater scope to manage symptoms effectively.

## Figures and Tables

**Table 1:** Baseline characteristics of the study population (*n* = 4886).

Variable	Category	*n* (%)
Gender	Male	2218 (45.42%)
	Female	2665 (54.58%)
Periodontitis	No	3851 (78.82%)
	Yes	1035 (21.18%)
Hypertension	No	4274 (87.47%)
	Yes	612 (12.53%)
Angina pectoris	No	4550 (93.12%)
	Yes	336 (6.88%)
Chronic ischemic heart disease	No	4717 (96.54%)
	Yes	169 (3.46%)
Atrial fibrillation	No	4822 (98.69%)
	Yes	64 (1.31%)
Heart failure	No	4824 (98.73%)
	Yes	62 (1.27%)
Cerebral infarction	No	4860 (99.47%)
	Yes	26 (0.53%)
Arterial occlusion/stenosis of precerebral arteries	No	4832 (98.89%)
	Yes	54 (1.11%)
Other cerebrovascular disease	No	4821 (98.67%)
	Yes	65 (1.33%)
Atherosclerosis	No	4791 (98.06%)
	Yes	95 (1.94%)
Non-toxic goiter	No	4772 (97.67%)
	Yes	114 (2.33%)
Type 2 diabetes mellitus	No	4786 (97.95%)
	Yes	100 (2.05%)
Obesity	No	4768 (97.58%)
	Yes	118 (2.42%)
Disorders of lipoprotein metabolism	No	4590 (93.94%)
	Yes	296 (6.06%)
Pneumonia	No	4818 (98.61%)
	Yes	68 (1.39%)
Gastroesophageal reflux disease	No	4811 (98.47%)
	Yes	75 (1.53%)

Note: Data are presented as counts with percentages for categorical variables. The study population included 4886 individuals, with demographic and clinical characteristics detailing the prevalence of periodontitis and associated comorbidities, including cardiovascular diseases, cerebrovascular conditions, metabolic disorders, and other systemic diseases.

**Table 2:** Proportional hazards test for equality of survival functions using Cox regression-based likelihood ratio (LR) Test.

Variable	Category	Observed Events	Expected Events	Relative Hazard	LR Chi^2^	*p*-Value
Gender	Male	104	92.75	1.13	2.27	0.132
	Female	126	137.25	0.92		
Periodontitis	No	185	195.42	0.95	3.44	0.064
	Yes	45	34.58	1.31		
Hypertension	No	201	217.92	0.95	18.29	**<0.001**
	Yes	29	12.08	2.47		
Angina pectoris	No	211	222.53	0.97	13.02	**<0.001**
	Yes	19	7.47	2.6		
Chronic ischemic heart disease	No	212	225.52	0.97	23.88	**<0.001**
	Yes	18	4.48	4.16		
Atrial fibrillation	No	221	227.48	0.99	10.12	**0.002**
	Yes	9	2.52	3.62		
Heart failure	No	221	227.55	0.99	10.51	**0.001**
	Yes	9	2.45	3.73		
Cerebral infarction	No	212	228.07	0.98	49.4	**<0.001**
	Yes	18	1.93	9.84		
Arterial occlusion/stenosis of precerebral arteries	No	219	227.75	0.98	17.77	**<0.001**
	Yes	11	2.25	5.01		
Other cerebrovascular disease	No	213	227.43	0.98	36.32	**<0.001**
	Yes	17	2.57	6.91		
Atherosclerosis	No	213	227.15	0.98	33.31	**<0.001**
	Yes	17	2.85	6.22		
Non-toxic goiter	No	214	226.57	0.98	24.84	**<0.001**
	Yes	16	3.43	4.82		
Type 2 diabetes mellitus	No	218	226.94	0.98	15.25	**<0.001**
	Yes	12	3.06	4		
Obesity	No	218	227.43	0.98	18.54	**<0.001**
	Yes	12	2.57	4.79		
Disorders of lipoprotein metabolism	No	210	222.57	0.97	15.18	**<0.001**
	Yes	20	7.43	2.76		
Pneumonia	No	219	225.92	0.98	8.2	**0.004**
	Yes	11	4.08	2.73		
Gastroesophageal reflux disease	No	208	224.93	0.97	32.01	**<0.001**
	Yes	22	5.07	4.53		

Notes: The table presents results from the Cox regression-based likelihood ratio (LR) test for equality of survival functions, assessing the impact of each covariate on neurodegenerative disease (NDD) risk. Observed and expected event counts reflect the distribution under the null hypothesis. Relative hazard represents the risk ratio between groups. The LR chi^2^ statistic and *p*-value indicate whether a covariate significantly influences survival. The LR test was used instead of the log-rank test due to tied event times, ensuring a robust comparison of survival distributions. Bold values indicate statistical significance, with *p* < 0.05.

**Table 3:** Multivariable Weibull regression model for neurodegenerative disease risk.

Variable	Hazard Ratio [95% CI]	*p*-Value
Age (years)	**1.05 [1.04–1.07]**	**<0.001**
Sex (Male vs. Female)	0.84 [0.65–1.10]	0.204
Periodontitis	**1.43 [1.02–1.99]**	**0.037**
Hypertension	1.40 [0.89–2.19]	0.143
Angina pectoris	0.96 [0.54–1.68]	0.875
Chronic ischemic heart disease	1.45 [0.82–2.56]	0.206
Atrial fibrillation	0.95 [0.46–1.94]	0.887
Heart failure	1.09 [0.52–2.28]	0.824
Cerebral infarction	**4.81 [2.90–7.96]**	**<0.001**
Arterial occlusion/stenosis of precerebral arteries	1.30 [0.67–2.52]	0.435
Other cerebrovascular disease	**2.09 [1.20–3.65]**	**0.01**
Atherosclerosis	**1.81 [1.03–3.17]**	**0.038**
Non-toxic goiter	**2.99 [1.71–5.24]**	**<0.001**
Type 2 diabetes mellitus	**1.95 [1.05–3.62]**	**0.034**
Obesity	**2.58 [1.37–4.86]**	**0.003**
Disorders of lipoprotein metabolism	0.84 [0.48–1.46]	0.534
Pneumonia	**1.96 [1.05–3.64]**	**0.035**
Gastroesophageal reflux disease	**2.82 [1.77–4.51]**	**<0.001**

Note: The table presents hazard ratios (HR) with 95% confidence intervals (CI) derived from the Weibull regression model assessing the association between various covariates and the hazard of developing NDD. A hazard ratio greater than 1 indicates an increased risk, while values below 1 suggest a protective effect. Statistically significant associations (*p* < 0.05) are highlighted with bold format. Age was analyzed as a continuous variable, while all other covariates were binary (present vs. absent) with the absence of each given covariate acting as a reference category. The model accounts for the underlying time-to-event distribution, providing adjusted hazard estimates for each covariate.

## Data Availability

The datasets generated and/or analyzed in the current study are available from the corresponding author upon reasonable request.
